# Challenging problems of applying microalgae for aquaculture environment protection and nutrition supplementation: A long road traveled and still a far way to go

**DOI:** 10.3389/fbioe.2023.1151440

**Published:** 2023-03-03

**Authors:** Qian Lu, Yujie Lu, Limin Yang

**Affiliations:** ^1^ School of Grain Science and Technology, Jiangsu University of Science and Technology, Zhenjiang, China; ^2^ School of Life Sciences, Jiangsu University, Zhenjiang, China

**Keywords:** microalgae, aquaculture, water environment, nutrition, sustainability

## 1 Introduction

It has been widely recognized that the development of traditional aquaculture in many countries is challenged by the severe contamination of water, abuse of medicines or antibiotics, and shortage of high-quality fishmeal (Wilfart et al., 2023). Therefore, the improvement of water quality in aquaculture, reduction of medicines or antibiotics usage, and substitution of fishmeal by alternative protein source have been intensively studied in recent years and considered as a potential way to upgrade the aquaculture industry (López-Pedrouso et al., 2020; Lulijwa et al., 2020). Under this situation, microalgae, some of which are enriched with protein, contain various immune-enhancing components, and have nutrients-removing capacity, are emerging into the limelight owing to their potential applications in aquaculture (Lu et al., 2021).

Some key opinions of previous studies focusing on algae-based aquaculture environment protection are listed as follows. Firstly, microalgae perform well in adsorbing heavy metals and removing nutrients (ammonia, phosphorus, etc.), creating a favorable aquaculture environment and safeguarding fish against toxicity (Alagawany et al., 2021). Secondly, some microalgae enriched with bioactive components, such as astaxanthin, phycocyanin, and polyunsaturated fatty acids, are proven to be effective against some aquaculture pathogens and able to improve immune response of fish (Alagawany et al., 2021). Thirdly, protein-rich microalgae can be added in diet to substitute fishmeal (Deng et al., 2021). Positive effects of microalgae-based diet on fish productivity, reproductive performance, and meat quality were revealed (Dineshbabu et al., 2019; Altmann et al., 2020). However, some problems which hinder the application of microalgae in aquaculture were neglected by previous studies. Herein, we would like to give constructive comments on these issues and provide an in-depth discussion of the application of microalgae for aquaculture environment protection and nutrition supplementation. It is expected that our comments and discussions can prevent the overly optimistic attitudes towards the practical application of microalgae in aquaculture environment protection and nutrition supplementation, spurring researchers to find out practically-feasible solutions to these challenging problems.

## 2 Problems of applying microalgae in aquaculture

### 2.1 Assimilation of heavy metals and nutrients by microalgae

As documented by previous studies, due to the negative charge on the surface of algal cells, microalgae have good capacity of adsorbing heavy metals in water environment (Leong and Chang, 2020; Alagawany et al., 2021). Hence, with the addition of living or non-living microalgae, a portion of heavy metals in aquaculture environment can be fixed. In addition, microalgae growth continuously consumes some nutrients, such as ammonia, phosphorus, and organic carbon, in water body (Li et al., 2021). Water environment with lower concentrations of nutrients can be more favorable to the growth of farmed fish.

In aquaculture, nevertheless, it may not be a practically-feasible strategy to safeguard fish against the toxicity of heavy metals by using microalgae. There are two problems that have not been solved yet. Firstly, microalgae-based heavy metals adsorption is a dynamic process (Richards and Mullins, 2013). With the death and decomposition of algal cells, heavy metals adsorbed or absorbed by microalgae will return to the water environment. Therefore, microalgae should be harvested from water environment after the process of heavy metal adsorption. Otherwise, dead microalgae with heavy metals will become a pollutant source, challenging the survival of farmed fish. In aquaculture activity, however, it is a very complex, expensive, and time-consuming process to continuously harvest microalgae from the water body of aquaculture (Li et al., 2021). Secondly, since microalgae are natural diets of herbivorous fish, heavy metals adsorbed on algal cells will enter fish body. In the long-term aquaculture activity, a large amount of algal biomass can be consumed by herbivorous fish, resulting in the accumulation of heavy metals in fish meat and blood. It is too hard to prevent the uptake of microalgae by herbivorous fish in aquaculture activity (Beal et al., 2018).

Similar problems are encountered in the microalgae-based nutrients removal in aquaculture system. Firstly, after the death and decomposition of algal cells, nutrients assimilated by microalgae would return to water environment. For example, Alagawany et al. cited the publication of Chuntapa et al. (2003) to discuss the contribution of *Spirulina* to nutrients removal and water quality improvement in the culture system of black tiger shrimp (*Penaeus monodon*) but neglected the fact that *Spirulina* was semicontinuously harvested from aquaculture tanks to prevent the cell death and decomposition (Chuntapa et al., 2003; Alagawany et al., 2021). In a real-world application, dramatic changes of weather can suddenly cause massive death of algal cells, challenging the wide use of microalgae for nutrients removal in aquaculture activity. Secondly, due to the consumption of HCO_3_^−^, microalgae growth is accompanied with the dramatic increase of pH in water environment. It was reported that the growth of *Spirulina* can increase the pH to around 12, creating an alkaline environment and threatening fish survival (Lu et al., 2017).

Based on the discussion above, microalgae may not effectively eliminate the threats of heavy metals and other pollutants to farmed fish although the bioabsorptive capacities of microalgae are excellent.

### 2.2 Unfavorable components of microalgae

According to previous studies, essential amino acids, polyunsaturated fatty acids (PUFAs), vitamins, and natural pigments, contained in microalgae, including cyanobacteria, green algae, and diatom, are regarded as high-value components for aquaculture activity (Tocher, 2015; Ahmad et al., 2020). However, some unfavorable components, particularly cell wall fiber and anti-nutritional factors (ANFs), contained in microalgae were neglected by Alagawany et al. and many previous studies (Alagawany et al., 2021). If the negative effects of algae-containing unfavorable components on fish growth are taken into consideration, we do not think the overly-optimistic attitudes towards microalgae-based fish diet are reasonable.

Since cellulose and hemicellulose are structure-supporting compositions of algae cell wall, microalgae contain high content of fibers. For example, the content of crude fiber in *Spirulina* and *Chlorella* (dry weight) could reach 4.07% and 9.43%, respectively (Seghiri et al., 2019; Metsoviti et al., 2020). As the fishmeal is substituted by microalgae, fiber content in fish diet would increase. It has been widely recognized that high fiber content in fish diets could increase the passage rate of feed in fish gut, reducing nutrient availability and feed digestibility (Ju et al., 2012; Ansari et al., 2021). As a result, feed conversion ratio is increased while fish growth rate is reduced.

In addition to fibers, some ANFs contained in microalgae may pose a threat to farmed fish when microalgae are utilized in fish diet. Since 1980s, a variety of anti-nutritional factors have been identified in microalgae (Cannell et al., 1988; Ishihara et al., 2006). For example, through the experiment of screening 300 freshwater and 200 marine eukaryotic algae, and 70 freshwater and 10 marine cyanobacteria, Cannell et al. (1988) obtained 39 species of algae containing protease inhibitors (Cannell et al., 1988). Therefore, the intake of microalgae containing ANFs may pose a threat to the digestive metabolisms of fishes.

In aquaculture activity, detrimental effects of excessive amount of algae biomass in diet on fish growth have been widely reported. For example, Deng et al. (2021) found that when the inclusion level of *Chlorella* in fish diet reached 25%, weight gain and length increase of fish (*Micropterus salmoides*) started to decrease (Deng et al., 2021). In this case, the substitution ratio of microalgae in fish diet must be kept very low and algal biomass can not be employed to replace fishmeal for farmed fish. In addition, based on literature review, Alagawany et al. reported that fishes have very different responses to the diet supplemented with microalgae (Alagawany et al., 2021). El-Sheekh et al. (2014) reported that diet with supplementation of 75% *Spirulina* had positive effects on the growth performance of red tilapia while Kim et al. (2013) observed the negative effects of *Spirulina*-supplemented diet on weight gain, growth rate and feed conversion ratio of parrot fish culture when the supplementation level was higher than 5% (Kim et al., 2013; El-Sheekh et al., 2014). In our view, this is mainly attributed to the different responses of fish species to the microalgae-based diet. Therefore, given the potential detrimental effects of microalgae on the growth of some fish species, in some cases, algal biomass may not be a good nutrition source for farmed fish.

### 2.3 High cost of algae biomass production

In many previous studies, microalgae biomass is regarded as a cheap protein source for aquatic animals. At present, microalgae can not be regarded as a cheap protein source. According to the statistical data released by Index Mundi, in the past 5 years (2017–2022), the prices of fishmeal in global market fell in a range of 1,360–1640 USD per metric ton (1.36–1.64 USD per kilogram) (IndexMundi, 2022). However, the production costs of algae are higher than 4-5 EUR per kilogram (Norsker et al., 2011; Guccione et al., 2014). In the model of co-production of *Chlorella*-based protein and renewable fuel, unit price of algal protein even reached 7.15–7.27 USD per kilogram (Karan et al., 2022). Compared to other alternative protein sources, such as soybean meal (418 USD per metric ton) and cornmeal (178 USD per metric ton), microalgae have no price advantages (García-Ulloa et al., 2017). Hence, Guccione et al. emphasize that the commercialization of microalgae as food/feed commodity is not mature yet (Guccione et al., 2014).

In recent years, a lot of efforts have been devoted to reducing microalgae biomass cost for the production of affordable feed ingredient. For example, flue gas was adopted to provide low-cost carbon source for *Chlorella* cultivation and dry algal powder was incorporated in formulated fish diet at an inclusion level of 15% (Yadav et al., 2020). The cost estimation shows that the cost of algae was 0.54 USD per kg, which is lower than that of fishmeal (0.84 USD per kg). Thus, the formulated fish diet with microalgae showed a marked reduction in total cost by 0.16 USD (26.1%) as compared to conventional feed (Yadav et al., 2020). Besides, other advanced technologies, such as the substitution of artificial medium with waste stream and the production of microalgae on biofilm, have also been studied as a possible path to obtaining low-cost microalgae biomass (Lu et al., 2015; Hu et al., 2021). With the installation of photovoltaic equipment, cultivation of algae in desert with sufficient solar energy is also regarded as an advanced technology to reduce the cost of algae production (Pruvost et al., 2019; Rasheed et al., 2022).

However, it must be noted that there is a huge gap between academic research and industrial application. In the practice, due to the limitations of actual situations, some advanced technologies developed in lab research to reduce microalgae biomass cost have not been commercialized yet. For example, in the regions or countries with low level of industrialization, it is not feasible to obtain a huge amount of flue gas or waste stream to cultivate low-cost microalgae biomass for fish diet production. Also, wastewater-based algae production for feed ingredient production has very strict requirements on bacteria controlling, nutrients profile balancing, and water microenvironment regulation, which may not be fully mastered by many feed production companies (Li et al., 2021). Therefore, it is not absolutely reasonable to regarded microalgae biomass as a cheaper protein source than fishmeal for fish diet production. In the future, to further promote the utilization of microalgae for fish feeding, more practically-feasible technologies should be developed to reduce the cost of algae biomass.

## 3 Summary and recommendations

The application of microalgae to construct eco-friendly aquaculture is an emerging trend in recent years. According to our research experiences and literature review, there are some problems challenging the application of microalgae in aquaculture activity. Firstly, if microalgae can not be harvested timely from aquaculture system, assimilated heavy metals and nutrients may be released back to water environment with the death and decomposition of algal cells. As a result, the survival of farmed fish will be seriously challenged. Fortunately, with the algae immobilization technologies (microalgae biofilm, fungal-algal pellets, microbial mat, etc.), suspended algal cells can be harvested timely. Secondly, microalgae contain a variety of unfavorable components, including cell wall fibers and ANFs, which may pose a threat to the digestive metabolisms of farmed fish. In a real-world application, microalgae with lower content of unfavorable components should be screened. Besides, novel process technologies, such as cellulase-based fiber decomposition, fermentation, and genetic techniques-based algal strains improvement, can be employed to remove those unfavorable components, attenuating the negative effects of diet with high inclusion level of microalgae on farmed fish. Thirdly, in some cases, microalgae-based fish diet has no price advantage over traditional fish diet owing to the high production cost of algal biomass. To further lower the cost of algal biomass, affordable and readily available nutrient sources, particularly food processing effluent without toxic components and CO_2_-rich flue gas, could be explored (Cheah et al., 2015; Gupta and Pawar, 2018).
